# A disturbed balance between blood complement protective factors (FH, ApoE) and common pathway effectors (C5a, TCC) in acute COVID-19 and during convalesce

**DOI:** 10.1038/s41598-022-17011-7

**Published:** 2022-08-11

**Authors:** Krzysztof Laudanski, Tony Okeke, Kumal Siddiq, Jihane Hajj, Mariana Restrepo, Damodar Gullipalli, Wen-chao Song

**Affiliations:** 1 Department of Anesthesiology and Critical Care, The University of Pennsylvania, JMB 127, 3620 Hamilton Walk, Philadelphia, PA 19146 USA; 2 Leonard Davis Institute for Health Economics, The University of Pennsylvania Colonial Penn Center, 3641 Locust Walk, Philadelphia, PA 19104 USA; 3 School of Biomedical Engineering, Science and Health Systems, Drexel University, Philadelphia, PA USA; 4 College of Arts and Sciences, Drexel University, Philadelphia, PA USA; 5 School of Nursing, Widener College, Chester, PA USA; 6 College of Arts and Sciences, The University of Pennsylvania, Philadelphia, PA USA; 7 Department of Systems Pharmacology and Translational Therapeutics, The University of Pennsylvania, Philadelphia, PA USA

**Keywords:** Viral infection, Acute inflammation

## Abstract

A complement effect on homeostasis during infection is determined by both cytotoxic (activate complement component 5 (C5a) terminal cytotoxic complex (TCC)), and cytoprotective elements (complement factor H (FH), as well as apolipoprotein E (ApoE)). Here, we investigated the gap in knowledge in their blood milieu during SARS-CoV-2 infection with respect to the viral burden, level of tissue necrosis, and immunological response. 101 patients hospitalized with a PCR-confirmed diagnosis of COVID-19 had blood collected at H1 (48 h), H2 (3–4 Days), H3 (5–7 days), H4 (more than 7 days up to 93 days). Pre-existing conditions, treatment, the incidence of cerebrovascular events (CVA), a history of deep venous thrombosis (DVT) and pulmonary embolism (PE), and mortality was collected using electronic medical records. Plasma C5a, TCC, FH, and ApoE were considered as a complement milieu. Tissue necrosis (HMGB1, RAGE), non-specific inflammatory responses (IL-6, C-reactive protein), overall viral burden (SARS-CoV-2 spike protein), and specific immune responses (IgG, IgA, IgM directed αS- & N-proteins) were assessed simultaneously. C5a remained elevated across all time points, with the peak at 5–7 days. Studied elements of complement coalesced around three clusters: **#0** (↑↑↑C5a, ↑↑TCC, ↓↓ApoE), **#1** ↑C5a, ↑TCC, ↑↑↑FH); **#2** (↑C5a, ↑TCC, ↑FH, ↑↑↑ApoE). The decline in FH and ApoE was a predictor of death, while TCC and C5a correlated with patient length of stay, APACHE, and CRP. Increased levels of C5a (Δ = 122.64; p = 0.0294; data not shown) and diminished levels of FH (Δ = 836,969; p = 0.0285; data not shown) co-existed with CVA incidence. C5a correlated storngly with blood RAGE and HMGB1, but not with viral load and immunological responsiveness. Remdesivir positively affected FH preservation, while convalescent plasma treatment elevated C5a levels. Three clusters of complement activation demonstrated a various milieu of ApoE & FH *vs* C5a & TCC in COVID-19 patients. Complement activation is linked to increased necrosis markers but not to viral burden or immune system response.

## Introduction

Acute infection with severe acute coronavirus syndrome coronavirus 2 (SARS CoV-2) manifests via multiple clinical presentations with varying degrees of clinical severity^1–3^. The highly heterogeneous natural history of COVID-19 is attributed to interactions between several homeostatic components and viral pathogens^4–7^. The loss of complement self-regulation is considered a pivotal factor in COVID-19 outcomes as coagulation abnormalities, increased necrosis, vasculitis, and monocyte overactivation are all hallmarks of COVID-19^8–13^.

The activation of the complement system is initiated via three different pathways coalescing around the formation of soluble C5a and C5b^8^. The latter partakes in forming the total cytolytic complex (TCC) as the terminal effector pathway^8,14^. C5a is important for granulocyte migration, anaphylaxis, and immune system activation^15,16^. TCC has direct bactericidal action but can also induce hemolysis and tissue damage^17^. The over-production of C5a and TCC is linked to unfavorable outcomes in several infections, including COVID-19, as well as coagulation and autoimmune illnesses^8,11,13,18,19^.

C5a and TCC actions need to be moderated to reduce collateral damage^8,20,21^. Interestingly, blood regulatory factors of the complement system were not studied in COVID-19 despite playing a role in regulating mechanisms that are particularly exaggerated in this illness^8,12,13,22^. Complement factor H (FH) is essential in inhibiting complement activation via necrotic tissue, a common occurrence in COVID-19^21,23–29^. Tissue damage and necrosis are often signified by the release of heat shock proteins, the high-mobility group box 1 protein (HMGB1), and other intercellular mediators, triggering complement activation and immunological response via several mechanisms, including the receptor for advanced glycation end products (RAGE)^30–32^. Consequently, FH is critical in dampening the initial triggers of complement activation^10,14,21,28^. FH also downregulates the activation of leukocytes directly and via pentraxin-related mechanisms^20,28,33–37^. Apolipoprotein E is critical in transporting cholesterol to the neuronal tissue and in the regulation of the complement cascade^38–40^. Namely, ApoE interferes with sC1q and TCC formation and suppresses monocyte activation, synergizing its moderating role with FH^38,39^. In addition, both ApoE and FH influence the dissociation rate of the C3 and C5 convertases^21^. Finally, ApoE and FH exhibit anti-complement regulatory properties through direct synergistic interactions with the cytolytic complement components and the non-complement checkpoints of inflammation^41,42^. Consequently, aberrations in the balance between them and C5a and TCC may underlie complement dysregulation and unfavorable clinical outcome^43,44^.

Initially, the complement system is activated secondary to viremia triggering necrosis and apoptosis directly^6^. Viremia and tissue damage trigger immune system response, which is heterogeneous. Finally, the resolution of the inflammation begins, but the post-infection recovery process is variable, particularly in COVID-19 patients^45^. Clinical symptoms of COVID-19 may be different at each stage, and the dynamics of the complement balance may account for clinical heterogeneity. For example, COVID-19 results in an increasingly hypercoagulable state, cardiomyopathy, cerebrovascular events (CVA), and kidney failure, albeit not in all patients^3,11,18,46–48^. Coagulation abnormalities are frequently linked to coagulation factor consumption (thrombocytopenia, prolonged international normalized ratio (INR), ↑↑↑d-dimer), vasculitis, and inflammation (↑↑↑IL-6, ↑↑↑ferritin, ↑↑↑CRP). The overlap between them and COVID-19 symptoms is significant^4,11,13^. These abnormalities may mediate secondary to the abnormal milieu of C5a, TCC, FH, and ApoE^6,8,11,22^. However, most studies have examined the complement effector with a short follow-up^4,6,22,44,45^. Data are conflicting as both persistence and normalization of complement activation have been demonstrated at 24 and 30 days^22,45^. However, most of the studies focused on hospitalized patients with severe diseases. Long-term follow-up is particularly important as the persistence of complement abnormal milieu may result in the emergence of cerebrovascular events and delayed organ failure.

COVID-19 treatments have very different anti-viral mechanisms of action^49^. One could hypothesize that implementing proven anti-COVID-19 therapy focused on reducing the late “cytokine storm” modulates how early viremia and necrosis affect excessive complement activation^8,19,21,22,50–58^. This would be a predominant mechanism for steroids and convalescent plasma^54,55,59^. On the other hand, plasma contains both inactive complement effectors and protective mechanisms, potentially augmenting or inhibiting complement activation itself^54,55^. Alternatively, if complement activation is secondary to the cytotoxic and necrosis activation, remdesivir should profoundly impact complement^57,58^. In any case, very little data are available regarding the effect of these treatments on the complement activation milieu despite the potential to target treatments for specific complement imbalances^13,59^.

This study investigates the gap in knowledge of the complement factors milieu during acute infection with SARS-CoV-2 and recovery instead of focusing on a particular complement component. We specifically addressed C5a and TCC activation in the protective levels of FH and ApoE. Both FH and ApoE moderated the effector potential of C5a and TCC as common pathways and ways to activate complement can occur during SARS-CoV-2. We analyzed the longitudinal complement changes and correlated them to the levels of immune system activation and viral burden. Finally, we assessed the effect of various treatments, such as remdesivir, convalescent plasma, and steroids, on the complement factor milieu.

## Methods

### Consent

Institutional Review Boards of the University of Pennsylvania approved the study (#813913) and conducted it according to the 2003 Helsinki Declaration. Written informed consent was obtained from all enrolled patients. This prospective single-center study enrolled a convenience sample of patients (n = 101) admitted to the hospital with a PCR-positive, acute COVID-19 infection between March and September 2020.

### Sample procurement

Blood was drawn when feasible once the patient consented. Time points were divided into less than 48 h (H1), 2–3 days (H2), 3–7 days (H3), and more than 7 days up to a total time of 93 days (H4) from the onset of hospitalization (Supplemental Figure S1).

Blood was collected in Vacutainer tubes (BD, Franklin Lakes, NJ) and put on ice. Downstream processing of biological specimens was done following BSL-2 enhanced standards. Plasma was collected after spinning the line at 1000×*g*, 10 min, 4 °C within 3 h of collection. Aliquot plasma was stored at − 80 °C. The plasma was inactivated via the incubation of 100 µl of plasma with 5% Tween-20 (Bioworld, Baltimore, MD) for 20 min at room temperature for biomarkers. In the case of complement assay, the plasma was inactivated by adding Triton X-100 and subjecting the sample to 55 °C for 1 h.

### Measures of complement activation

To detect human C5a levels in inactivated plasma samples, 96-well plates were coated with an anti-human C5a antibody neo-epitope (Clone C17/5; Biolegend, San Diego, CA) at a final concentration of 2 μg/mL in PBS at 37 °C for 1 h. Plates were blocked with 1% BSA in PBS for 1 h at RT. Following washes with PBS containing 0.05% Tween-20, the plates were incubated with diluted plasma samples in blocking solution at RT for 1 h. After washing, the plates were incubated with biotinylated anti-human C5a mAb (Clone G25/2; Biolegend, San Diego, CA) at a final concentration of 1 μg/mL in blocking solution at RT for 1 h, washed, and incubated with avidin or streptavidin conjugated to horseradish peroxidase (BD, San Jose, CA) in blocking solution at RT for 1 h. After the final washing, the plates were developed with HRP substrate for 3 min. The reaction was stopped with 2 N H_2_SO_4,,_ and the plate was read at 450 nm in a microplate reader. Recombinant hC5a (Hycult; Wayne, PA) was used as the standard.

To detect soluble TCC levels in inactivated plasma samples, 96-well plates were coated with an anti-human TCC mAb neo-epitope (Clone AE11; Santa Cruz; San Jose, CA) at a final concentration of 2 μg/mL in PBS at 37 °C for 1 h. These plates were then blocked with 1% BSA in PBS for 1 h at RT. Following washes with PBS, containing 0.05% Tween-20. The plates were incubated with diluted plasma samples in blocking solution at RT for 1 h. After washing, the plates were incubated with biotinylated anti-human C5 mAb (QDC5 inhouse) at a final concentration of 1 μg/mL in blocking solution at RT for 1 h, washed again, and incubated with avidin or streptavidin conjugated to horseradish peroxidase (BD; San Jose, CA) in blocking solution at RT for 1 h. After the final washing, the plates were developed with HRP substrate for 3 min. The reaction was stopped with 2 N H_2_SO_4,_ and the plate was read at 450 nm in a microplate reader. C5b-9 Complex (Complement Tech; Alameda, CA) was used as the standard.

### Assessment of the viral burden, tissue destruction, and inflammation biomarkers

The viral burden was measured via plasma level of S-protein, using a commercially available kit (Raybiotech, Norcross, GA). The level of tissue destruction was assessed with a plasma level of HMGB-1 with ELISA (Aviva System Biology, San Diego CA). ELISA was read at 450 nm with correction for 560 nm absorbance using a commercially available reader. The specific immuno-globulin response to SARS-CoV-2 was assessed by an evaluation of blood immunoglobulins against proteins S&N with the help of a commercial kit (Raybiotech, Norcross, GA). The absorbance OD value was subtracted from albumin-coated plates and referenced against the standard curve. Inflammation was measured with ICRP and sRAGE (Theromofisher, Waltham, MA), while D-dimer (Theromofisher, Waltham, MA) was utilized as a measure of coagulation abnormalities and inflammation. Multiplex kits were measured on a MagPix machine (Luminex; Austin, TX). IL-6 was measured using O-link technology.

### Collection of clinical data

Electronic medical records (EMR) were employed to collect demographic and medical data on all enrolled participants, including patients' self-determined race and ethnicity. Values for ferritin, INR, and platelets were obtained from laboratory data. Acute Physiology And Chronic Health Evaluation II (APACHE II) was calculated within one hour (APACHE_1hr_) and at 24 h after admission (APACHE_24hrs_)^60^. The burden of chronic disease was calculated using the Charlson Comorbidity Index (CCI)^61^. The severity of the illness was determined by a Marshall's Organ Dysfunction Score (MODS) and a Sequential Organ Failure Assessment (SOFA)^62^. Organ failure was defined according to RIFLE criteria or the Glue Grant framework^63,64^. Diagnoses of deep venous thrombosis (DVT), pulmonary embolism (PE), and stroke were extracted from the providers’ notes in EMR. Survival was determined at 28 days.

Treatments with hydroxychloroquine, remdesivir, convalescent plasma, or steroids were highly protocolized per hospital policy, according to the FDA at the time of the study^52,54,57,58,65^. Per healthcare provider notes, steroid treatments were determined to engage any intravenous or oral glucocorticoid compounds to treat COVID-19 pneumonia.

### Statistical analysis

The Shapiro–Wilk W test and distribution plots were used to test the normality and distribution of variables. Parametric variables will be expressed as mean ± SD and compared using *t*-Student. For non-parametric variables, median (M_e_) and interquartile ranges (IR) will be shown with the U-Mann–Whitney statistic employed to compare such variables. ANOVA was calculated for parametric variables with multiple discrete values using Scheffe’s posthoc test. Paired contrast was used when applicable. Correlational momentum was calculated as a *r* Pearson value. Only correlations above r ≥ 0.25 and with p ≤ 0.05 were included. The regression analysis was done using stepwise methods. Clustering analyses were done, using k-means to assess the distance of similarity. The Kaplan Meier survival curves (KMC) were generated using the *survival* and *survminer* packages in R. A *p*-value less than 0.05 will be considered statistically significant for all tests based on the hypothesis. Statistical analyses will be performed with the SPSS 26 (IBM, Endicott, NY), and in R (R Core Team, 2014), and figures will be generated, using the *ggplot2* and *ggpubr* packages.

### Ethics approval and consent to participate

The study was conducted according to the guidelines of the Declaration of Helsinki and approved by the Institutional Review Board of the University of Pennsylvania (#813913; approved 03.02.2020).

## Results

### Characteristics of the sample and their relationship to complement factors

The demographics and clinical data for a total of 101 patients enrolled in the study are presented in Table 1. Individuals over the age of 60 had statistically significant differences in plasma C5a levels (C5a_<60 years_ = 192.7 ± 189.56 *vs* C5a_>60 years_ = 282.6 ± 221.22; p = 0.012) and lower expressions of TCC (TCC_<60 years_ = 0.79 ± 1.62 *vs* sTCC_older_ = ; TCC_>60 years_ = 1.4 ± 2.67; p = 0.089). Gender differentiated individuals in respect to C5a (sC5a_male_ = 204.64 ± 178.12 *vs* sC5a_female_ = 294.31 ± 239.93; p = 0.016) and TCC level (sTCC_male_ = 0.8383 ± 1.80 *vs* sTCC_female_ = 1.5696 ± 2.77; = 0.078). Race did not differentiate C5a, TCC, ApoE, and FH blood levels (data not shown).The burden of pre-existing disease did not correlate with TCC or ApoE, but there were weak yet significant correlations with C5a and FH (data not shown).

**Table 1:** Demographic and clinical characteristics of the studied samples.

**Demographics (101 patients)**				
Age [X ± SD]	58.36 ± 18.29			
**Age**				
Below 60 [%]	44.6%			
Over 60 [%]	54.5%			
**Gender**				
Male [%]	60.4%			
Female [%]	39.6%			
Not reported [%]	0%			
**Race**				
Caucasian/Hispanic Latino [%]	26.73%			
Black [%]	63.37%			
Other/Asian/unknown [%]	9.9%			
**Pre-existing conditions**				
Charleston comorbidity index [X ± SD]	3.49 ± 2.95			
Acute coronary syndrome [%]	4.95%			
Congestive heart failure [%]	12.87%			
Peripheral vascular disease [%]	6.93%			
Cerebrovascular disease/Transient ischemic attack [%]	9.9%			
Dementia [%]	3.96%			
Chronic obstructive pulmonary disease [%]	11.88%			
Connective tissue disease [%]	2.97%			
Peptic Ulcer Disease [%]	2.97%			
Liver Disease [%]	1.98%			
Diabetes mellites [%]	35.64%			
Hemiplegia [%]	3.96%			
Chronic kidney disease [%]	24.75%			
Solid Tumor [%]	10.89%			
Leukemia [%]	0.99%			
Lymphoma [%]	9.9%			
Acquired immunodeficiency syndrome [%]	0%			
**Smoker**				
Smoker [%]	0.99%			
Former smoker [%]	9.9%			
Non-smoker [%]	56.44%			
Vaper [%]	0%			
Mortality [%]	15.84%			
Stroke [%]	5.66%			
Length of stay [X ± SD]	17.7 ± 26.65			
Intensive care Unit [%]	49.5%			
Intubated [%]	32.67%			
Extra corporeal membrane oxygenation [%]	8.91%			
APACHE admission + 1 h [X ± SD]	10.72 ± 7.65			
APACHE admission + 24 h [X ± SD]	10.76 ± 7.18			

Organ failures	H1 (%)	H2 (%)	H3 (%)	H4 (%)
Central nervous system failure [%]	8.91	4.95	6.93	6.93
Cardiovascular system failure [%]	26.73	12.87	17.82	11.88
Respiratory failure [%]	35.64	34.65	25.74	14.85
Renal failure (acute kidney injury) [%]	22.77	13.86	14.85	7.92
Liver failure [%]	42.57	26.73	23.76	10.89

### Longitudinal analysis of complement activation

**Figure 1:**
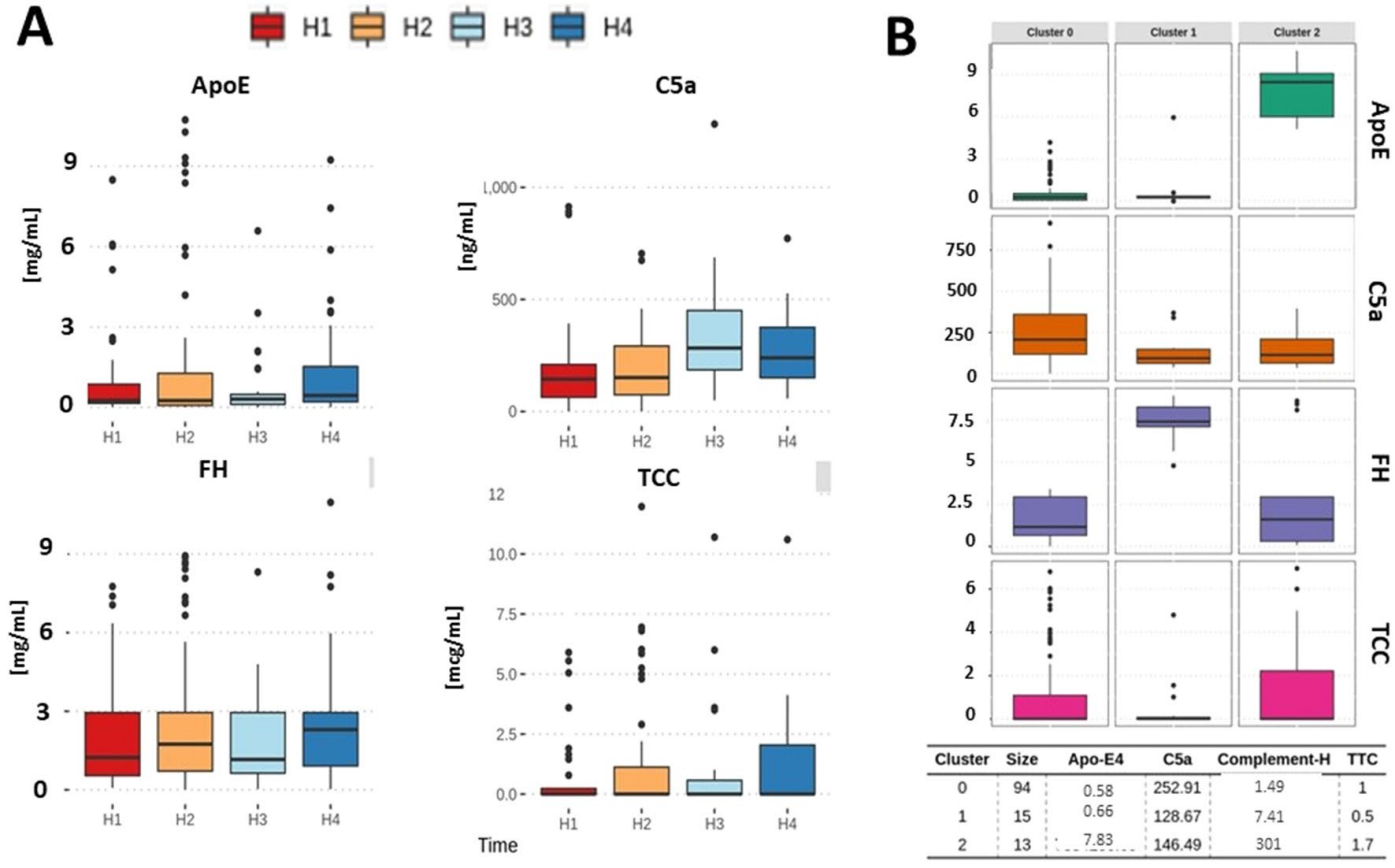
Distribution of complement factors across different time points (**A**) demonstrating the increase in C5a at H3 time point. The variability in ApoE and FH was significantly less. Elements of complement milieu associated themselves across all time points along with three different clusters (**B**) when all time points were analyzed. The majority of samples demonstrated a depletion of protective factors vs reactive component of complement (cluster #0). Cluster #1 was characterized by elevated FH and cluster32 by ApoE.

TCC, ApoE, and FH exhibited statistically non-significant longitudinal variability, but only C5a continued to increase over time (U[3;138]  = 13,098; *p* = 0.044) with a peak at H3 (Fig. 1A). Only significant correlation among all studied complement factors was TCC with ApoE (*r*^2^ = 0.3; *p* = 0.00058). Three clusters of association were identified among studied complement factors, with ApoE and FH being the major determinants across all studied samples and time points (Fig. 1B). Cluster #0 was characterized by the highest C5a, pronounced TCC, and a low level of both ApoE and FH. It was the dominant cluster in terms of the number of cases (Fig. 1B). Cluster #1 was signified by the dominant activation of FH and the low activation of other markers (Fig. 1B). Cluster #2 was comprised of individuals with the highest activation of ApoE, while other markers were somewhat modestly activated (Fig. 1B). Inflammation markers. (IL-6, CRP, ferritin) or coagulation measurements (platelet, INR) between clusters were different across all clusters yet not statistically significant. However, d-dimer levels were elevated in cluster #0 when compared to cluster #1 (p = 0.043) and #2 (p = 0.0084). Also, IgG against S&N SARS-CoV-2 were elevated in cluster #0 (p = 0.022) as compared to #1 or #2. IgM levels against S&N SARS-CoV-2, IgA against S&N SARS-CoV-2 or spike protein were not different between clusters.

### Clinical correlates of the complement factors milieu

**Figure 2:**
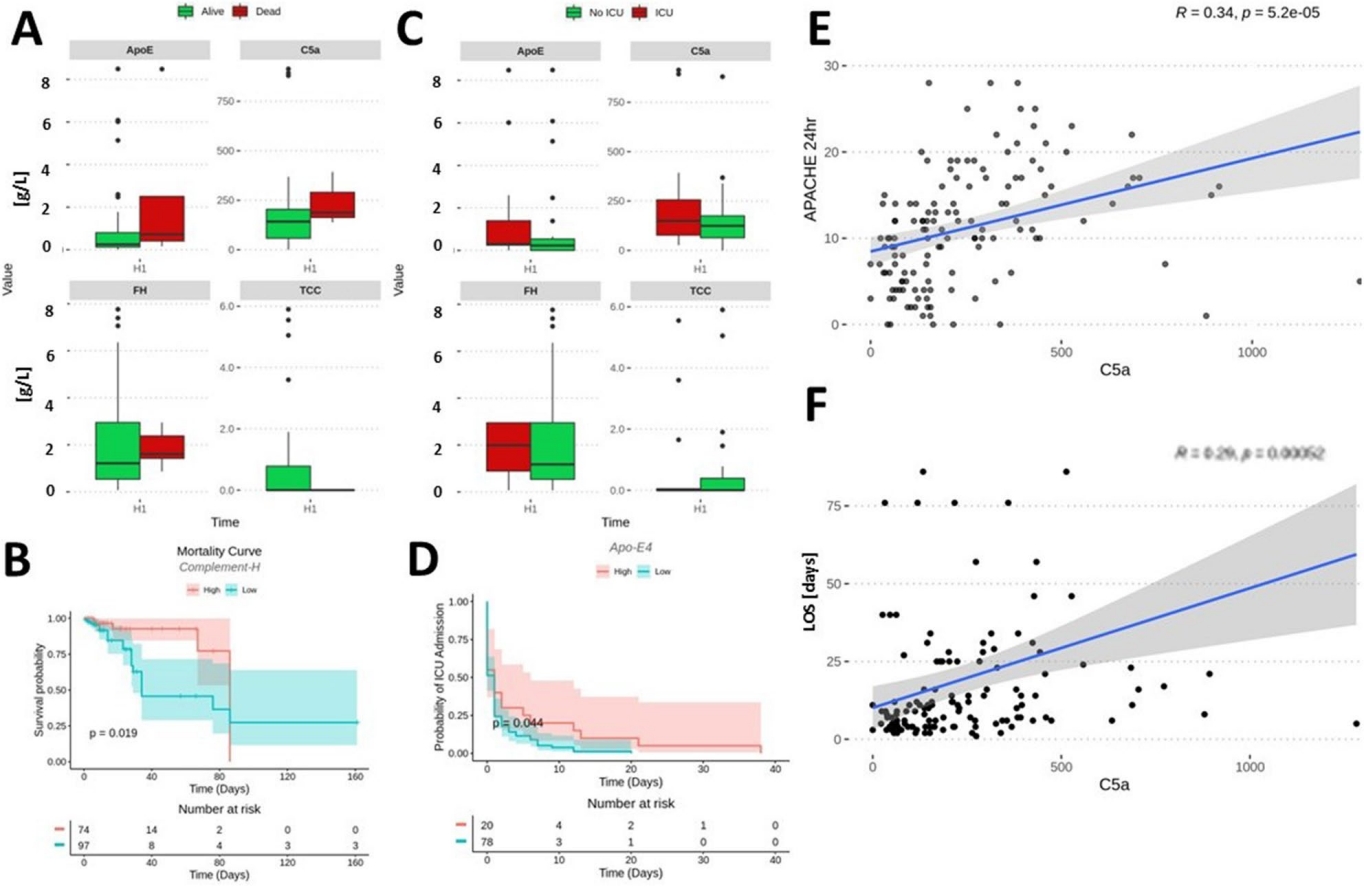
Distribution of complement markers at the first blood draw (H1) for alive *vs* dead (**A**) and No ICU vs ICU (**C**) patients when all time points were considered. Patient with blood complement factor below the mean value for all samples had a significantly increased risk of dying early in disease HR (95% CI 2.8 (1.1–7) (**B**). Depletion of ApoE below the mean from all blood samples also had a tendency to correlate with mortality HR (95% CI 0.97–2.8) (**D**). C5a activity correlated with APACHE II scores (**E**) and length of stay (LOS) (**F**).

Patients who died during hospitalization exhibited a significantly increased activity of C5a (U[138] = − 3.35; p = 0.006), while TCC, FH, and ApoE levels were similar to those at admission irrespective of patient survival (Fig. 2A). FH predicted mortality if the levels were low during the hospital stay (Fig. 2B). Admission to the ICU differentiated patients by their hospital admission level of C5a (U[138] = 2.986; p = 0.0264), but not TCC, ApoE, or FH (Fig. 2C). Low ApoE levels predicted ICU admission (Fig. 2D). There was a correlation between APACHE at admission and 24 h (Fig. 2E). LOS correlated significantly with plasma C5a levels only (*r* = 0.3; *p* = 0.00048) (Fig. 2F).

Finally, patients who were diagnosed with PE during the observation period had significantly increased levels of FH (Δ = 837952.64; p = 0.0385; data not shown), while differences across all other studied markers between patients with and without DVT were not significant. Patients diagnosed with stroke had increased levels of C5a (Δ = 122.64; p = 0.0294; data not shown) and diminished levels of FH (Δ = 836969; p = 0.0285; data not shown).

### The time evolution of the complement changes and response to viral infection

**Figure 3:**
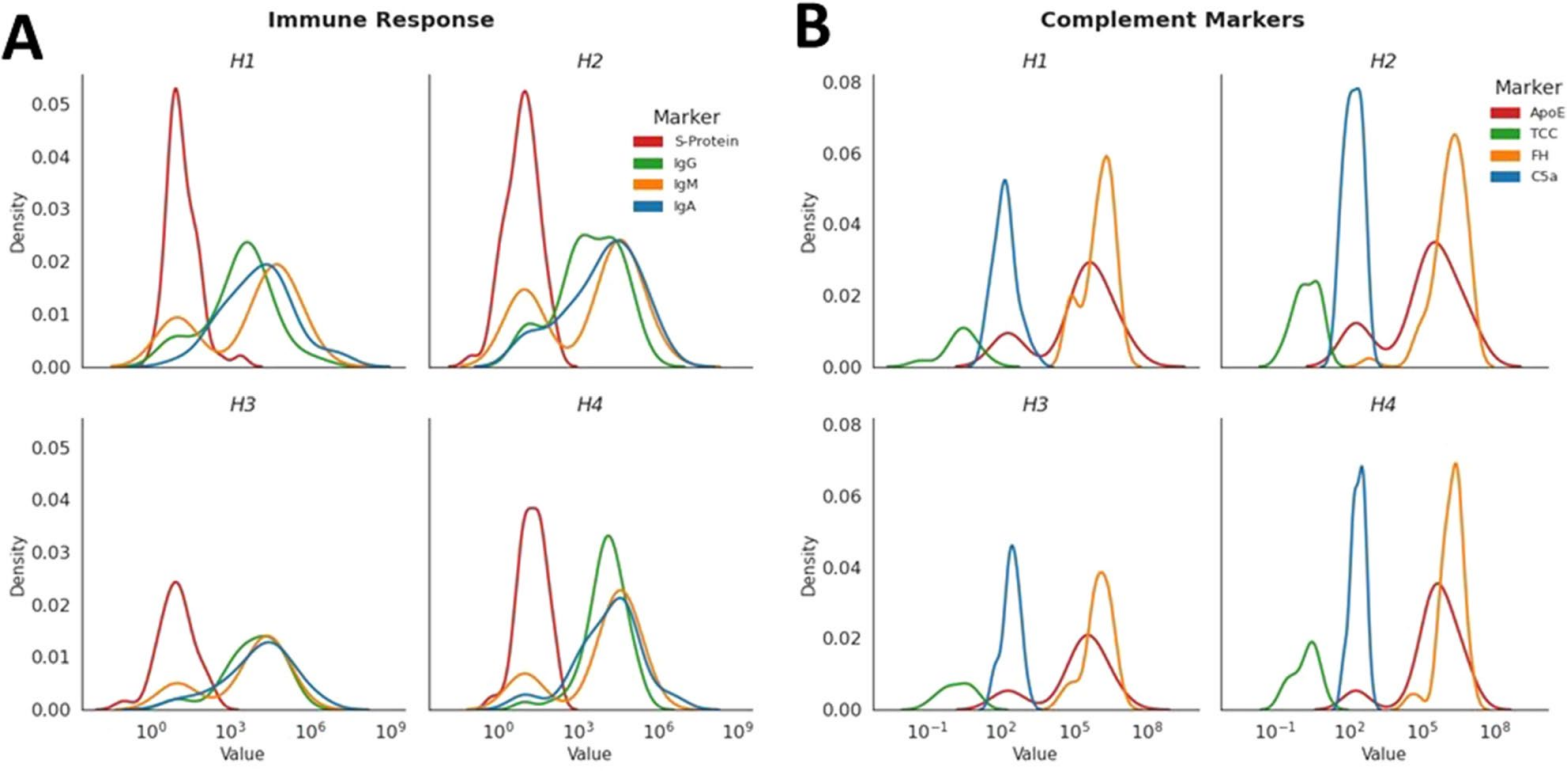
The distribution of immunoglobulin in response to viral protein levels demonstrated a diminishing level of S-spike protein and ongoing variability in immunoglobulins against S&N proteins (**A**). In addition, the complement evolution of time was signified by a bimodal increase in plasma C5a markers and elevated FH across all studied samples (**B**).

We found a significant variation across the levels of S-spike protein over time (Fig. 3A). Furthermore, antibodies against the S-spike protein changed significantly in our group (Fig. 3A). Neither change correlated with the measured activation of complement factors (data not shown), while the trajectory of complement components over time followed a different pattern (Fig. 3B). The H2 and H4 time points had significantly elevated C5a and TCC (Fig. 3B).

Among inflammation and infection markers, only C5a correlated positively with blood levels of S-spike proteins (*r* = 0.22; *p* = 0.0011) and negatively with CRP (*r* = − 0.17; *p* = 0.046) but not with other inflammatory markers. These correlations were very weak, even though statistically significant. Other elements of the complement system had non-significant correlations. The level of tissue necrosis during SARS-CoV-2 infection measured by blood HMGB1 correlated highly with the activity of TCC (*r* = 0.38; *p* = 0.00011) and FH (*r* = 0.29; *p* = 0.027). Correlations with non-specific markers of inflammation like sRAGE, CRP, IL-6, and ferritin were not significant.

### The effect of COVID-19 directed treatment on the complement milieu

**Figure 4:**
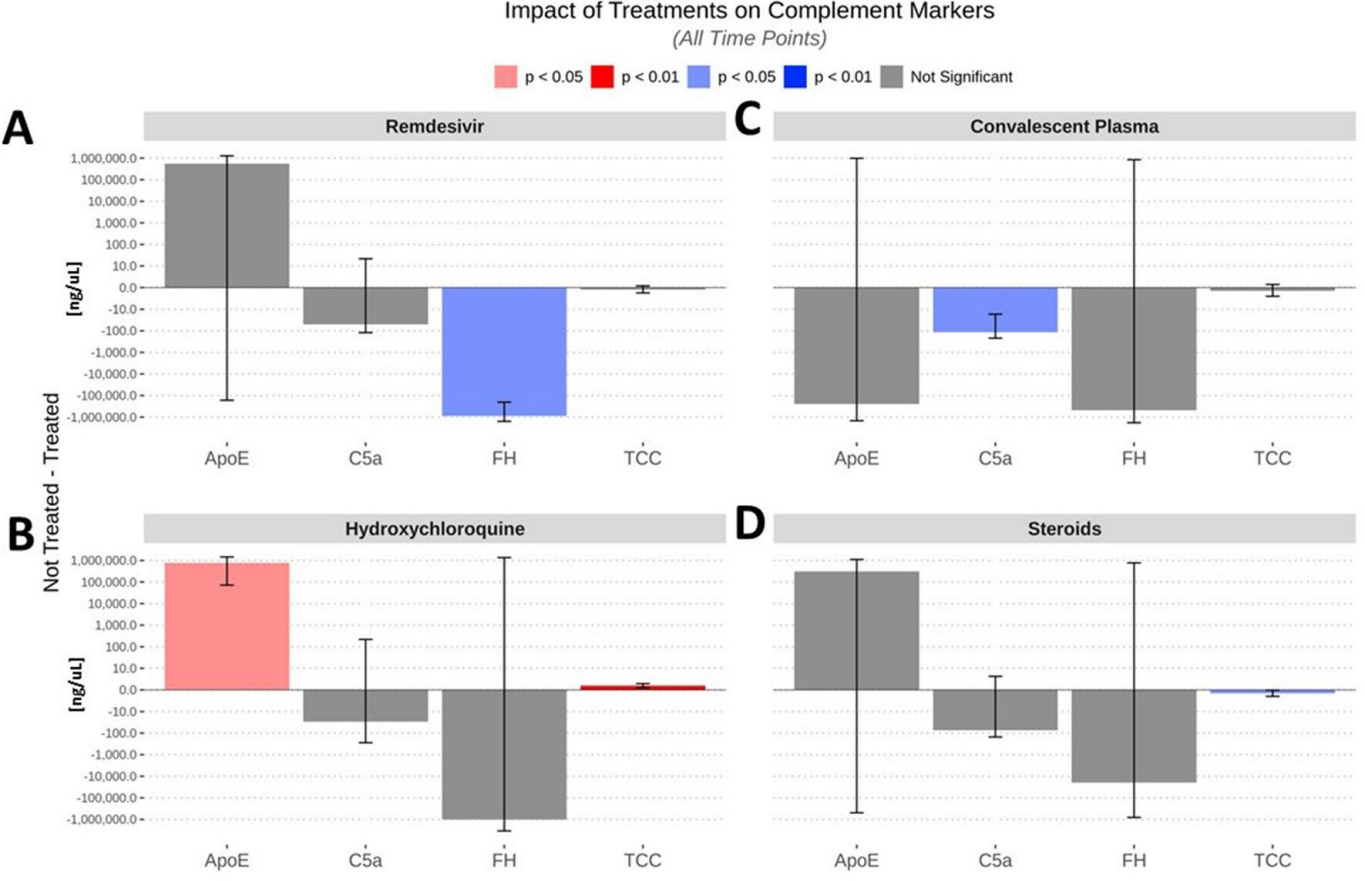
Effect of treatment with Remdesivir (**A**), Convalescent plasma (**B**), Hydroxychloroquine (**C**), and steroids (**D**) on complement marker levels. The data were analyzed across all time points and compared to times when patients were not treated with COVID-19 medications.

Finally, we assessed the effect of the treatments on the activation markers. Remdesivir resulted in a significant decrease in the levels of complement H (Fig. 4A). Convalescent plasma affected lower blood levels of C5a (Fig. 4B). Hydroxychloroquine increased the levels of TCC and ApoE, while steroids had precisely the opposite effect on TCC (Fig. 4C,D).

## Discussion

The unique finding of this study is the observation of a severe disruption in the balance between factors protecting from the excessive activation of the complement system *vs* activated cytolytic components. As demonstrated by cluster analysis, both ApoE and FH were severely depressed in several patients if their C5a and TCC were elevated. This approach to the data is well aligned with the current understanding of sepsis and COVID-19 resulting from system dysregulation^19,66^. Prior data demonstrated a similar clustering of phenotypes in terms of protective and damaging markers^9,22,28,37^. When focusing on a singular marker, the lack of progress from early studies on several critical care illnesses triggered more attention to a system analysis, where several biological components are considered^22,67,68^. This approach appreciates that every component of homeostasis consists of several parts that are complementary and partially redundant^13,67,68^. The complement system is not unique in this respect as its activation is guided by several non-specific stimuli, leading to two distinct yet complementary pathways with several regulatory components like FH and apoE^8,23^. Complement defense mechanisms are triggered quickly in response to several stimuli. They lack specificity but have expediency, which is critical for curbing the initial pathogen incursion. Subsequently, appropriate mechanisms need to be in place to curb prolonged complement activation^9,27,38,69^. Failure of these protective mechanisms has been demonstrated in several chronic diseases and in types of sepsis, but not in COVID-19^8^. The lack of the protective ability of ApoE and FH, rather than overactivation of C5a or TCC, was a predictor of death, suggesting that counterregulatory mechanisms are critical in recovery from COVI-19 and a critical care illness. This suggests that complement system dysregulation, instead of singular abnormalities, underpins COVID-19 pathology.

C5a increased over time to peek around seven days, while the high variability of TCC blood levels complicated the analyses. It was demonstrated previously that increased C5a and TCC are slow to return to baseline levels over 20–30 days^44^. This may reflect the slow resolution of complement activation, but the resolution was significantly prolonged in our study. This may link C5a to the emergence of long COVID-19, yet further data are critical to test the validity of this hypothesis. We noticed an increased level of C5a and diminished blood FH in patients experiencing cerebrovascular events. This may indicate that prolonged complement milieu abnormality of may be linked to prolonged events. The size of our sampling allows for designing a study that addresses the question of prolonged complement aberration and its link to CVA excess. However, patients enrolled in the study at and after day seven may be more severely ill due to enrollment bias, even though this was not reflected in our clinical data as several of our patients were discharged home.

This would be a bias shared by other studies retaining more sick patients in the follow-up. It is also possible that the C5a peak represents the natural evolution of the complement response after an illness. Though several studies have looked into C5a activation during viral, none conducted serial measurements to assess the deactivation timeline for C5a or TCC^70^. Also, the literature is focused on the harmful effect of C5a and TCC, while complement deficiencies are also linked to unfavorable clinical outcomes during infection. Interestingly, the inability to resolve sepsis early is related to poor survival^8,19,21,66^.

Several markers of COVID-19 severity correlated with C5a and TCC, but not with protective elements of complement activation. This suggests that COVID-19 severity aligns with cytolytic elements as part of the response to pathogen intrusion, while the inability to resolve the activation is related to demise. A correlation between C5a and HMGB1 and sRAGE suggests that necrosis and apoptosis are more related to the activation of the elements of the common complement pathway. In fact, complement is triggered by the release of necrotic DAMPs directly and via IgG. This process is predominant early in the illness and linked to the emergence of symptoms. We did observe some correlation with the APACHE score, a measure of symptom severity at admission to the hospital. If the DAMP and necrosis continue to be released, then activation of the complement will be protracted, especially in cases where protective elements are being exhausted. Considering that FH can bind and insulate necrotic tissue from complement initiators, its depletion would be particularly important. In fact, we demonstrated a subpopulation of the patient with depletion of FH, but the size of the group made any clinical correlations very speculative. The initial activation will trigger a profound and vigorous immune system response associated with the emergence of symptomatic illness. Consequently, the correlation between physical symptoms of infection and initial activation seems justified. Over time, the ongoing inflammation will exhaust the protective elements, leading to demise.

The disconnect between markers of complement activation, immune system activation, and viral load suggests that complement activation evolves somewhat independently. None of the markers correlated with levels of S-spike protein or immunoglobulins A, M, or G against this viral protein^3,8^. The time dynamics of the complement were significantly different from markers of viral burden. In contrast, complement activation has been described mostly in the context of apoptotic processes and the presence of several danger-associated activation patterns like DNA or glycogenotic surfaces^21,25,29,31,32,41,43,70^. This suggests that cellular destruction would drive complement activation, especially if the complement protective markers are insufficient^8,9,50^. Correlation with RAGE and HMGB-1 may reflect that both markers are related to non-specific activation due to excessive necrosis and apoptosis, or are the DAMP, respectively^50,69^. Immune system activation is driven by several factors during COVID-19. Consequently, a direct link between immune activation, complement, and illness is not surprising. Prior data demonstrated that COVID-19 immune activation is diverse, even early in the disease, and is not entirely dependent on viremia^71^. However, our data suggest that initial tissue destruction driven by early host–pathogen interaction may be the driver of complement interaction.

Our studied cohort's analysis of treatment strategies demonstrated the effect of convalescent plasma and remdesivir on cytoprotective elements but not on the cytolytic ones. This is the first study to provide such a piece of evidence to date. Both remdesivir and convalescent plasma is approved to treat moderate-to-severe COVID-19, while steroids are mostly utilized in patients undergoing mechanical ventilation^58,72,73^. In our study, steroids resulted in borderline changes in TCC. However, remdesivir treatment co-existed with the preservation of FH. Therefore, one could hypothesize that remdesivir dampens the cytolytic effect of SARS-CoV-2, reducing the release of necrotic material^13,21,50^. The subsequent activation of cytolytic complement components will be less, leading to a diminished need to control it and thus preserving FH and Apo4. Though we demonstrated the cellular-level relationship between cellular destruction and complement components, we did not observe correlations between viral burden and FH or Apo4. Finally, C5a changed upon a patient receiving convalescent plasma, but it is unclear if the measured C5a was from the patient or donor^6,44,54^. Our data suggest that elevated C5a can persist for over seven days. Others suggested similar, or even longer, observations^6,44^. Therefore, C5a could be coming from donors with an incomplete resolution of complement activation.

Though our study demonstrated three novel observations, its results should be considered thought-provoking instead of ultimate demonstrations. First, our patient sample was captured early in the pandemic and represented a wide variety of patients in terms of COVID-19 severity. However, there is an inherent bias at the H3 time point that reflects data from more severe patients, as the patients who recovered or had minimal symptoms were discharged before that time. On the other hand, our H4 population captured several patients in the convalescent phase. Some recovered from COVID-19 and may represent long-haulers^74,75^. This may explain why several markers were elevated at H4 even though the H3 time point was almost nominal, excluding C5a.

Additionally, having both patients fully recovered or still recovering from COVID-19 resulted in a bimodal distribution of several data at H4, thereby significantly limiting the statistical sensitivity of our analysis when using longitudinal statistical contrasts. However, the cluster analysis and relatively large sample size allowed for focusing the study on the composition of the markers instead of on longitudinal analysis. Local complement activation may bear more on organ failure and unfavorable clinical outcomes than blood levels. This was well described in the case of the involvement of the complement in acute lung illness and chronic kidney failure^7^. However, our study did not address this problem as we measured the global activation of complement. Finally, the effect of treatment modalities is challenging to assess as the samples were collected early during the pandemic. Although the applications of remdesivir and steroids were highly protocolized, treatment with convalescent plasma is challenging to quantify as the plasma's composition and biological activity were not routinely assessed.

## Conclusions

Three clusters of complement activation can be identified, demonstrating an imbalance between Apo4 and FH, leading to unopposed activation of C5a and TCC. The evolution of the complement response seems to correlate more significantly with markers of cellular destruction than viral load and components of inflammatory response. Remdesivir treatment preserved FH levels.

## Supplementary Information

Supplementary Figure 1.

## Data Availability

The datasets used and analyzed during the current study are available from the corresponding authors on reasonable request.

## Abbreviations

C5a: Complement component 5 activated
TCC: Transmembrane cytolytic complex
FH: Complement factor H
ApoE: Apolipoprotein E
RAGE: Heceptor for advanced glycation end products
HMGB-1: High mobility group box protein 1
CRP: C-reactive protein
IgG: Immunoglobulin type G
