# Supplementary material for: A disturbed balance between blood complement protective factors (FH, ApoE) and common pathway effectors (C5a, TCC) in acute COVID-19 and during convalesce
Source: Sci Rep. 2022 Aug 11;12:13658. doi: 10.1038/s41598-022-17011-7 (PMC9366819; doi:10.1038/s41598-022-17011-7)

Supplemental Material

Supplemental Figure 1

Histogram showing the distribution of patient samples into different time points.

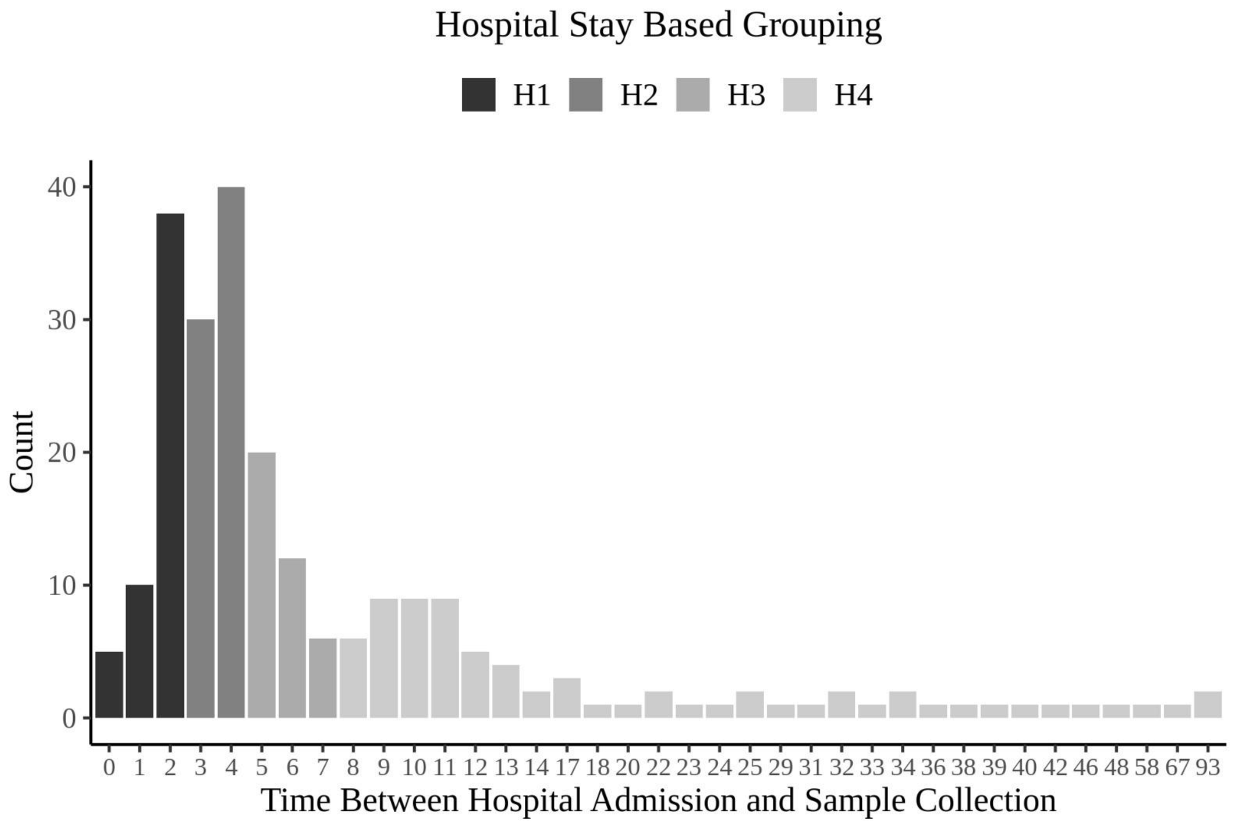

Supplement: Supplementary file 1 — Supplementary Figure 1. [file 41598_2022_17011_MOESM1_ESM.pdf]
